# Conformationally Selective 2-Aminotetralin Ligands Targeting the alpha2A- and alpha2C-Adrenergic Receptors

**DOI:** 10.1021/acschemneuro.3c00148

**Published:** 2023-04-27

**Authors:** Nicholas R. Fragola, Brittany M. Brems, Munmun Mukherjee, Meng Cui, Raymond G. Booth

**Affiliations:** ^†^Center for Drug Discovery, ^‡^Department of Pharmaceutical Sciences, ^§^Department of Chemistry & Chemical Biology, Northeastern University, 208, Mugar Life Sciences Building, 360 Huntington Avenue, Boston, Massachusetts 02115, United States

**Keywords:** 2-aminotetralin, α2A receptor, α2C receptor, molecular modeling, adrenergic receptor

## Abstract

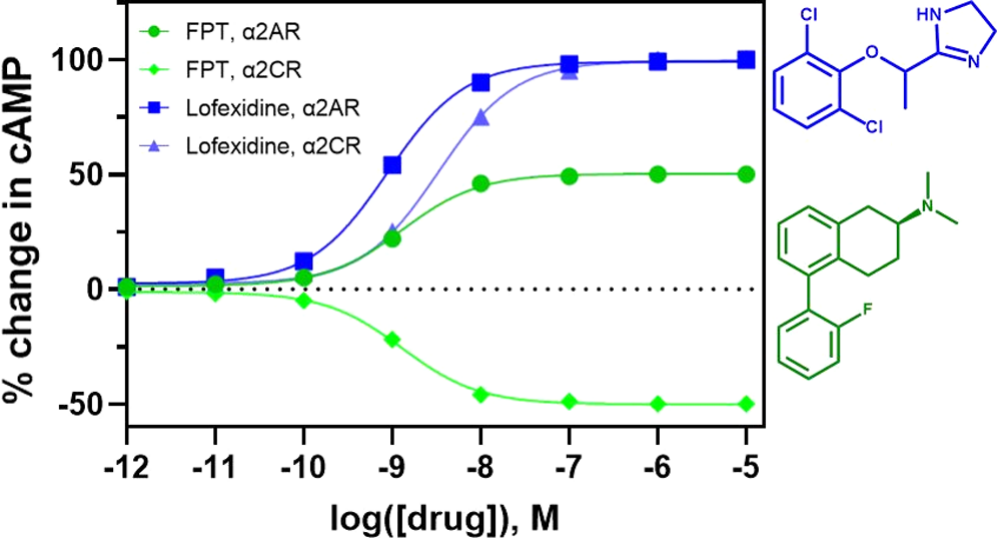

Many important physiological processes are mediated by alpha2A- and alpha2C-adrenergic receptors (α2Rs), a subtype of class A G protein-coupled receptors (GPCRs). However, α2R signaling is poorly understood, and there are few approved medications targeting these receptors. Drug discovery aimed at α2Rs is complicated by the high degree of binding pocket homology between α2AR and α2CR, which confounds ligand-mediated selective activation or inactivation of signaling associated with a particular subtype. Meanwhile, α2R signaling is complex and it is reported that activating α2AR is beneficial in many clinical contexts, while activating α2CR signaling may be detrimental to these positive effects. Here, we report on a novel 5-**s**ubstituted-2-**a**mino**t**etralin (5-SAT) chemotype that, depending on substitution, has diverse pharmacological activities at α2Rs. Certain lead 5-SAT analogues act as partial agonists at α2ARs, while functioning as inverse agonists at α2CRs, a novel pharmacological profile. Leads demonstrate high potency (e.g., EC_50_ < 2 nM) at the α2AR and α2CRs regarding Gα_i_-mediated inhibition of adenylyl cyclase and production of cyclic adenosine monophosphate (cAMP). To help understand the molecular basis of 5-SAT α2R multifaceted functional activity, α2AR and α2CR molecular models were built from the crystal structures and 1 μs molecular dynamics (MD) simulations and molecular docking experiments were performed for a lead 5-SAT with α2AR agonist and α2CR inverse agonist activity, i.e., (2*S*)-5-(2′-**f**luoro**p**henyl)-*N*,*N*-dimethyl-1,2,3,4-**t**etrahydronaphthalen-2-amine (FPT), in comparison to the FDA-approved (for opioid withdrawal symptoms) α2AR/α2CR agonist lofexidine. Results reveal several interactions between FPT and α2AR and α2CR amino acids that may impact the functional activity. The computational data in conjunction with experimental *in vitro* affinity and function results provide information to understand ligand stabilization of functionally distinct GPCR conformations regarding α2AR and α2CRs.

## Introduction

Noradrenergic signaling is the primary driver of the sympathetic nervous system (SNS),^1^ modulating physiological processes such as the fight-or-flight response, as well as regulating the activation of every major organ in the body, including in the central, cardiovascular, pulmonary, and circulatory systems.

The regulation of norepinephrine (NE) levels in different brain regions plays a critical role in several therapeutically relevant contexts, such as hypertension,^2^ attention-deficit/hyperactivity disorder (ADHD),^3,4^ and opioid withdrawal,^5,6^ as well as in working memory^7^ and executive function.^8^ NE levels are tightly regulated by α-2-adrenergic receptors (α2Rs), which include three subtypes: α2A, α2B, and α2C G protein-coupled receptors (GPCRs) that couple canonically to the Gα_i/o_ protein to modulate the activity of adenylyl cyclase (AC) and production of cyclic adenosine monophosphate (cAMP).^9^

The expressions of α2ARs and α2CRs are relatively high and distribution varies throughout the brain, while α2BRs have low central nervous system (CNS) expression and are localized primarily in the thalamus.^10,11^ The α2AR is the most widely distributed subtype, with particular density in the prefrontal cortex (PFC), hippocampus, and locus coeruleus (LC)^11,12^ as well as the amygdala,^13^ with the LC serving as the central origin of adrenergic output in the brain.^5^ The α2CR is primarily expressed in the striatum, hippocampus, and cortex.^12,14^ There is much interest in the study of α2ARs and α2CRs regarding their role in neuropsychiatric, neurological, and neurodevelopmental disorders.

**Figure 1:**
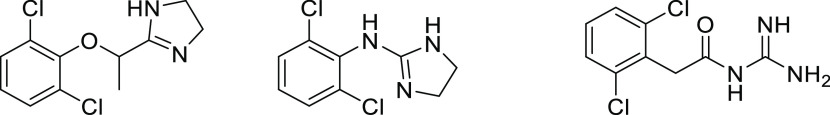
Lofexidine, clonidine, and guanfacine (from left).

For example, the nonselective α2A/2CR agonists clonidine and guanfacine (Figure 1) are approved to treat attention-deficit-hyperactivity disorder (ADHD)^8,15,16^ and the nonselective α2AR/α2CR agonist lofexidine is approved to treat opioid withdrawal symptoms.^17−19^ All three approved α2R agonists cause sedation.^19−22^ In addition, data from transgenic mice models suggest the cognitive^14,23^ and neurochemical^24,25^ benefits of these drugs may come from α2AR activation alone and α2CR activation may be deleterious to the benefits obtained from α2AR activation. In a study measuring cognitive performance, α2AR-knockout (KO) mice performed worse than wild-type (WT) mice, and there was no improvement in the KO mice performance with the administration of guanfacine.^3^ In another study, α2CR-KO mice showed better performance in cognitive tasks than WT mice, and the α2AR/α2CR agonist dexmedetomidine further improved α2CR-KO mice performance.^26,27^ Moreover, in a human clinical trial, an α2CR-selective antagonist (ORM-12741) was shown to increase episodic memory.^28^ Thus, there is evidence from rodent studies^26,27^ and human clinical trials that implies that there is neurotherapeutic benefit from the activation of the α2AR but not necessarily activation of the α2CR; in fact, the data from transgenic mouse studies suggest that activation of the α2CR may be deleterious with regard to cognition and behavior. Accordingly, development of compounds that are able to activate α2AR signaling while simultaneously inactivating α2CR signaling may show a superior neurotherapeutic profile. In general, however, discovery of drugs targeting α2ARs or α2CRs is confounded by the receptors’ high degree of overall homology (84% sequence overlap^29^), and they differ by only one amino acid in the orthosteric ligand binding pocket.^30,31^

The 5-**s**ubstituted-2-dimethyl**a**mino**t**etralin (5-SAT, Table 1) chemotype is a novel scaffold synthesized in our lab that has neurotherapeutic activities in mouse models.^32^ In particular, the 5-SAT FPT (Table 1) is an orally active analogue that improves repetitive behaviors, social behaviors, anxiety behaviors, and ameliorates seizures in mouse models of autism spectrum disorder (ASD),^32−34^ which often is comorbid with ADHD.^35^ Importantly, FPT did not impact locomotor activity in rodents, suggesting there may not be sedative effects and/or abuse liability.

**Table 1:**
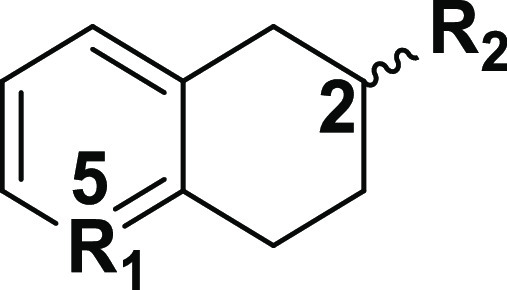

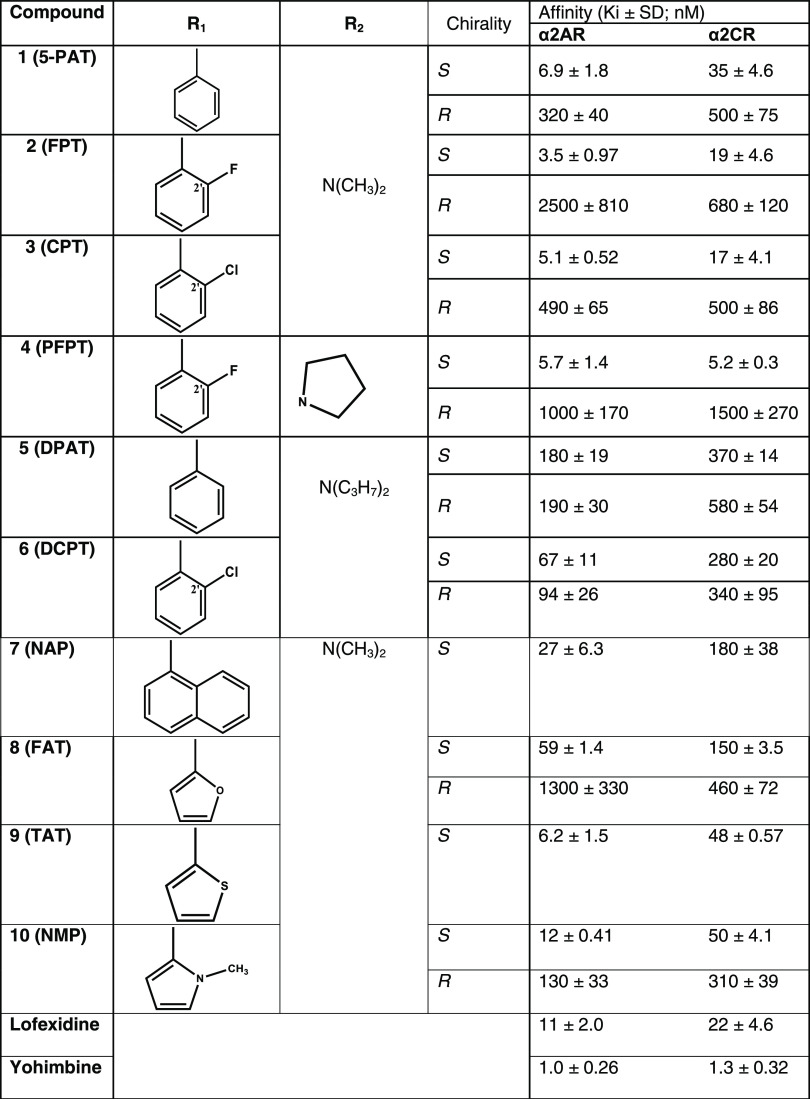
Binding Affinities of 5-Substituted-2-Aminotetralins at α2AR and α2CR

Here, we report syntheses of new 5-SATs and characterize their affinity and signaling (cAMP) function at α2ARs and α2CRs. Importantly, we document novel α2R pharmacology, i.e., the same 5-SAT that can activate α2AR signaling can inactivate α2CR signaling. We undertook computational chemistry and molecular modeling studies to study the interactions of FPT at the α2AR and the α2CR to infer molecular mechanisms of its unique pharmacology, i.e., α2AR agonism together with α2CR inverse agonism in the same small monovalent molecule. For comparison, we undertook similar studies using the approved (for opioid withdrawal) nonselective α2R agonist lofexidine.

## Results

### Affinities of 5-SATs at α2AR and α2CR

Affinities of 5-SATs were evaluated using radioligand competition binding assays with [^3^H]rauwolscine as the labeled ligand in comparison to the agonist lofexidine and the inverse agonist yohimbine (Table 1). Among 5-SAT analogues in Table 1 substituted with a C(2)-*N*,*N*-dimethylamine or -pyrrolidine (**1–4**, **7–10**), regardless of the C(5)-substituent, there is high binding preference (20–40-fold) for the *S*-enantiomer at α2ARs and α2CRs. In contrast, for the C(2)-*N*,*N*-dipropylamine analogues (**5**, **6**), regardless of the C(5)-substituent, there is only slight preference for *S*-enantiomer binding at the α2AR and α2CR and in general, the C(2)-*N*,*N*-dipropylamine analogues have lower affinity (10–25-fold) than the C(2)-*N*,*N*-dimethylamine and pyrrolidine analogues at both the α2AR and α2CR. The C(2)-*N*,*N*-dimethylamine and C(2)-*N*,*N*-dipropylamine analogues have 3–5-fold selectivity for binding the α2AR over the α2CR, but there is no binding selectivity for the C(2)-pyrrolidine analogue. Nevertheless, the C(2)-pyrrolidine compound has 3–30-fold higher affinity at the α2CR than any of the other analogues in Table 1.

Substituting the C(5)-position with larger aromatic groups (NAP) or heteroaromatic moieties (**8**–**10**) rather than phenyl greatly compromises affinity at the α2CR compared to the α2AR. Interestingly, there is 5–10-fold higher affinity of the TAT and NMP analogues compared to the FAT compound at α2AR. When C(5) is substituted with the large naphthyl aromatic moiety, affinity is compromised at the α2AR and even more so at the α2CR compared to when the 5-position is substituted with phenyl.

### 5-SAT α2AR- and α2CR-Mediated Modulation of cAMP Formation in Clonal Cells

Compounds in Table 1 with *K*_i_ ≤ 25 nM at the α2AR (**1–4**, **7**, **9**, **10**) were selected for assessment of functional activity at both the α2AR and α2CR. Cyclic adenosine monophosphate (cAMP) accumulation was measured via a modified LANCE Ultra cAMP time-resolved fluorescence resonance energy transfer (TR-FRET) immunoassay method, with the approved agonist lofexidine as a positive control and inverse agonist yohimbine as a negative control. Lofexidine was chosen as the reference ligand due to its ongoing use in *in vivo* studies evaluating 5-SATs for alleviation of opioid withdrawal symptoms.

**Figure 2:**
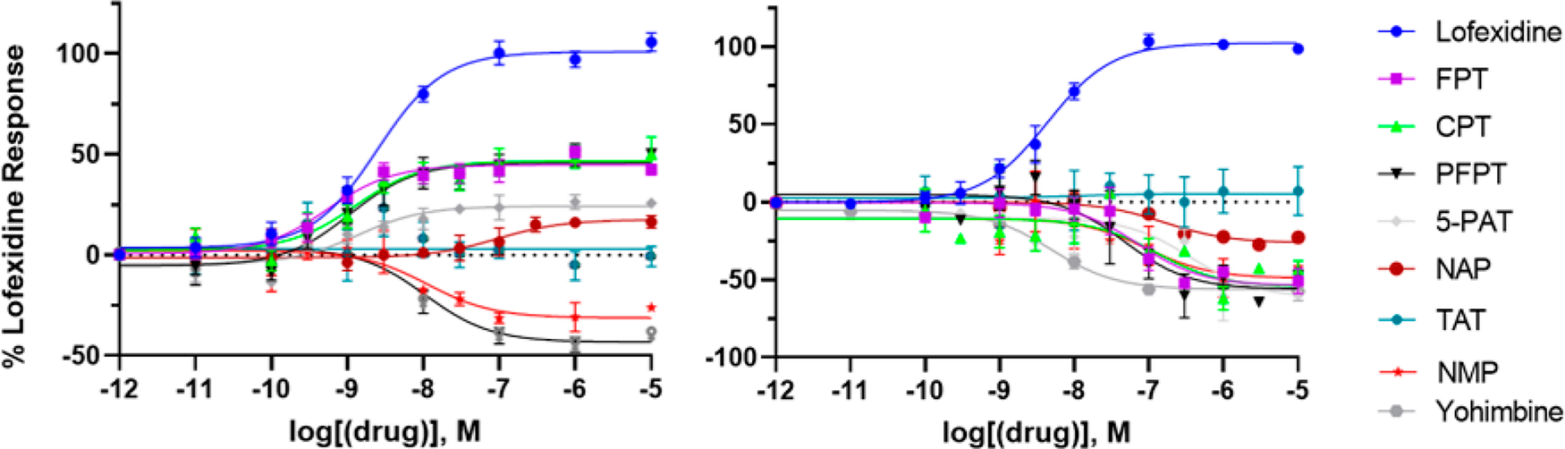
Comparative assessment of functional activity elicited by 5-SAT ligands in cells stably expressing the α2A-adrenoreceptor (left) and α2C-adrenoreceptor (right). The *y*-axis displays the unit of receptor activation, which was determined as percent activation relative to lofexidine. Percent change in cAMP over forskolin-stimulated baseline was determined, and then normalized as a function of the percent change stimulated by lofexidine. A minimum of five independent experiments were performed for each compound displayed, tested in triplicate in each experiment; data displayed represent the mean value across all experiments.

The 5-SATs evaluated that displayed agonist activity at the α2AR (**1**–**4**, **7**) showed inverse agonist activity at the α2CR (Table 2 and Figure 2). These results for the 5-SATs are unique compared to the functional activity for lofexidine, which is an agonist at both the α2AR and α2CR (Table 2 and Figure 2).

**Table 2:** Functional Activities of 5-SATs at α2AR and α2CR^a^

	α2AR		α2CR	
compound	EC_50_ or IC_50_^b^	*E*_max_ or *I*_max_^c^	EC_50_ or IC_50_^b^	*E*_max_ or *I*_max_^c^
**1** (5-PAT)	1.4 ± 0.23	24 ± 4.0	370 ± 1.9	–51 ± 3.4
**2** (FPT)	0.55 ± 0.15	45 ± 1.9	79 ± 1.7	–54 ± 4.7
**3** (CPT)	1.3 ± 0.18	47 ± 2.9	130 ± 2.4	–55 ± 6.7
**4** (PFPT)	1.1 ± 0.16	46 ± 3.0	48 ± 1.8	–56 ± 5.5
**7** (NAP)	82 ± 8.3	14 ± 4.3	260 ± 44	–26 ± 7.1
**9** (TAT)	NC^d^	NC^d^	NC^d^	NC^d^
**10** (NMP)	11 ± 0.26	–34 ± 3.8	92 ± 5.9	–48 ± 7.7
lofexidine	2.5 ± 0.11	100 ± 3.1	4.9 ± 1.2	100 ± 3.4
yohimbine	10 ± 0.34	–43 ± 5.4	5.0 ± 0.12	–56 ± 2.0

a Data shown are mean ± SD.

b EC_50_ is the concentration (nM) of ligand that decreases cAMP formation by 50% of ligand-elicited maximum; IC_50_ is the concentration (nM) of ligand that increases cAMP formation by 50% of ligand-elicited maximum.

c *E*_max_ and *I*_max_ are the percent maximum response of the ligand normalized to lofexidine.

d NC: no significant change from forskolin-established baseline signaling.

At the α2AR, when the C(5) of the 5-SAT scaffold is substituted with phenyl or 2′-halophenyl (**1–4**), analogues are significantly more potent (*F*_3,31_ = 33.65, *p* < 0.0001) than lofexidine. FPT (**2**) is significantly more potent than 5-PAT (**1**), CPT (**3**), and PFPT (**4**) (*F*_2,22_ = 48.69, *p* < 0.001) and, in fact, is the most potent α2AR agonist identified in these studies. When the C(5)-substituent of the 5-SAT scaffold is the large naphthyl moiety (NAP) rather than phenyl or 2′-halophenyl, potency is greatly reduced, e.g., NAP is 150-fold less potent than FPT. NMP is identified as an inverse agonist at the α2AR. For comparison, we evaluated the known inverse agonist yohimbine^36^ in our assay system and found that NMP and yohimbine display inverse agonist potencies at the α2AR that were not significantly different (*p* = 0.4829, unpaired *t*-test).

At the α2AR, the (2*S*)-*N,N*-dimethyl and (2*S*)-pyrrolidine-substituted 5-SATs with a 5-(2′-halophenyl) substituent (**2–4**) behave as partial agonists with roughly half the maximum efficacy of lofexidine (Table 2 and Figure 2), with no significant differences (*F*_2,22_ = 0.1511, *p* = 0.8607) in *E*_max_; when the 5-substituent is 5-phenyl (5-PAT), maximum efficacy is ∼25% compared to lofexidine. With a larger C(5) aromatic group, NAP efficacy is 14% of lofexidine. Interestingly, at the α2AR, the 5-(2′-*N*-methyl-pyrrole) NMP analogue (**11**) is an inverse agonist, increasing the accumulation of cAMP (*I*_max_ = 34%). The inverse agonist yohimbine was not significantly more efficacious as an inverse agonist than NMP (*p* = 0.2218, unpaired *t*-test). The 5-thienyl-substituted TAT displayed no significant change over baseline, suggesting a neutral antagonist profile.

At the α2CR, all of the 5-SATs tested are inverse agonists except the 5-thienyl analogue TAT, which appears to be a neutral antagonist like at the α2AR (Figure 2). The potency of (2*S*)-pyrrolidinyl analogue (PFPT) is highest and about 2-fold and 3-fold higher than other 5-SATs (FPT and CPT, respectively) with a 5-(2′-halophenyl) moiety; the potency of PFPT is about 9-fold higher than 5-PAT, which does not have a halogen on the C(5) phenyl moiety. The 5-(2′-halophenyl)-substituted 5-SATs had potencies roughly 10-fold less than that of reference inverse agonist yohimbine.

Additionally, at the α2CR, none of the inverse agonist efficacies of the 5-SATs are significantly different (*F*_4,13_ = 0.2909, *p* = 0.8787) except for NAP, which was modestly (roughly 2-fold) but significantly (*F*_5,15_ = 3.23, *p* = 0.0354) less efficacious. As was the case at the α2AR, TAT also appears to be a neutral antagonist at the α2CR. In comparison to reference inverse agonist yohimbine, efficacies of the 5-phenyl-substituted 5-SATs assessed (**1–4**) are not significantly different (*F*_4,14_ = 0.1740, *p* = 0.9481).

Due to its high potency at the α2AR juxtaposed against inverse agonist activity at the α2CR, as well as its encouraging performance in mouse models of autism^32−34^ and ongoing use in studies in nonhuman primates, FPT was selected for further analysis in molecular modeling studies in order to elucidate the molecular determinants for binding and function at the α2AR and α2CR.

### *In Silico* Docking of (*S*)-FPT and Lofexidine at α2AR and α2CR Molecular Models

**Figure 3:**
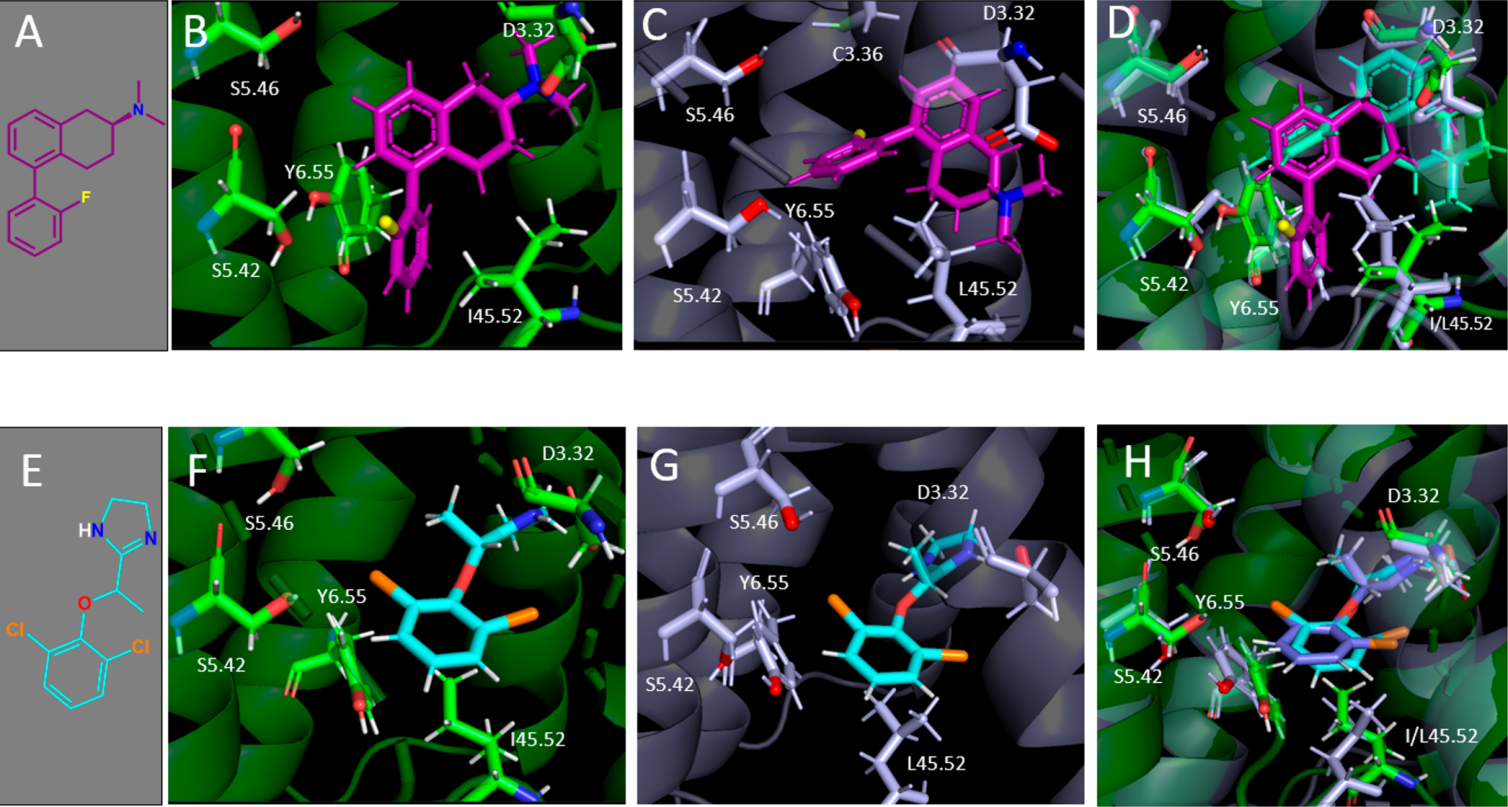
Molecular models and ligand docking. (A) 2-D structure of S-FPT. (B) *S*-FPT docked at the α2AR model. (C) *S*-FPT docked at the α2CR model. (D) Superimposed docks of *S*-FPT at α2AR (green) and α2CR (light blue) models. (E) 2-D structure of lofexidine. (F) Lofexidine docked at the α2AR model. (G) Lofexidin*e* docked at the α2CR model. (H) Superimposed docks of lofexidine at α2AR (green) and α2CR (light blue) models.

The unique opposing functional activities of the 2′-halogenated 5-SATs at the α2AR and α2CR prompted us to investigate the molecular determinants governing the unique functional profile. The α2AR and α2CR share a high degree of homology, with 84% sequence homology.^29^ The α2AR and α2CR ligand binding pocket amino acids for FPT and lofexidine are identical except for the extracellular loop 2 (ECL2) position 45.52, which is isoleucine (I) at the α2AR and leucine (L) at the α2CR.^30,31^ Molecular models were developed using the solved crystal structures of the α2AR^31^ and α2CR,^30^ and the binding poses of *S*-FPT and lofexidine were compared at both receptors (Figure 3).

At the α2AR model (Figure 3B), the dimethylamine moiety of FPT (presumably protonated at physiological pH) docks close (2.9 Å) to the D3.32 residue, likely forming an ionic bond characteristic of aminergic ligand interaction with Class A GPCRs.^37,38^ The FPT fluorophenyl moiety docks close (3.7 Å) to Y6.55, apparently forming π–π interactions as well as a halogen bond^39−41^ between the 2′-fluorine and the tyrosine hydroxyl. The fluorophenyl moiety also docks close (3.5 Å) to S5.42, apparently forming a halogen–hydrogen bond between the FPT 2′-fluoro moiety and the serine hydroxyl moiety.

At the α2CR model (Figure 3C) however, the FPT pose is rotated about 90° clockwise compared to the pose at α2AR. The FPT dimethylamine moiety still docks close (2.9 Å) to D3.32 and the fluorophenyl moiety appears to be close enough (2.9 Å) for π–π interactions with Y6.55, as noted for the α2AR dock. However, the 2′-fluorine moiety does not appear to interact with S5.42, though FPT may be close enough (4.0 Å) to the C3.36 sulfhydryl moiety to realize a halogen bond.

Figure 3D shows the superimposed docks for FPT at the α2AR and the α2CR. The major difference in binding modes of FPT at the α2AR and the α2CR appears to involve the ECL2 position 45.52, which protrudes into the binding pocket. For example, the α2CR L45.52 residue may impede the FPT 2′-fluorophenyl moiety interaction with S5.42 and Y6.55 (Figure 3C), leading to an FPT orientation that is shifted deeper into the pocket and rotated ∼90° relative to the conformation at the α2AR. The cyclohexyl region of the FPT tetrahydronaphthyl moiety does not appear to make productive binding interactions with I45.52 at the α2AR, whereas, there appear to be hydrophobic (e.g., van der Waals) interactions with L45.52 at the α2CR.

At the α2AR and α2CR models (Figure 3F,G), lofexidine binds in almost identical poses, as is clear from the superimposed docks in Figure 3H. For both the α2AR and α2CR models, the lofexidine imidazoline nitrogen (presumably protonated at the physiological pH) is close enough (about 2 Å) to the conserved D3.32 residue such that an ionic bond likely is realized, similar to FPT (Figure 3B,C). The centroids of the lofexidine dichlorophenyl moiety and the α2AR Y6.55 residue are close enough (about 3 Å) to realize π–π interactions (Figure 3F), as is also observed for the α2CR Y6.55 residue (Figure 3G), approximating a similar pose to FPT at the α2AR (Figure 3B). The lofexidine dichlorophenyl moiety also docks near (3.6 Å) α2AR S5.42 (Figure 3F), apparently forming a halogen bond with the S5.42 hydroxyl moiety, analogous to the fluorophenyl moiety of FPT at the α2AR (Figure 3B). The lofexidine dichlorophenyl moiety also may realize interaction (at a distance of 3.8 Å) with the α2CR S5.42 residue (Figure 3G)―this is in contrast to the FPT dock at α2CR (Figure 3C) where no interaction is realized. Another difference in the poses of lofexidine and FPT at the α2AR and α2CR is that the nonconserved residue at position 45.52 in ECL2 likely does not realize any productive binding interactions with lofexidine at either receptor. Finally, the oxygen linker moiety of lofexidine may also be stabilized by π interaction with nearby F6.51 (not shown in Figure 3), helping to stabilize the “aromatic cage” region of the receptor, i.e., residues F7.39, F6.51, Y7.43, and W6.48 that stabilize the binding pocket in the active conformation of the receptor,^30,31^ and which may account for the higher efficacy observed for lofexidine compared to FPT at both receptors.

### Molecular Dynamics Simulations of the (*S*)-FPT Interaction with α2AR and α2CR

In all class A GPCRs in the inactivated state, there is an ionic bond (lock) between the conserved R3.50 and D6.30 residues. During ligand-stabilized GPCR activation, there is disruption of this R3.50/D6.30 interaction, concomitant with outward movement of transmembrane domain (TM) 6.^42,43^ Thus, the R3.50/D6.30 distance can be measured as a proxy for receptor activation. Here, the R3.50/D6.30 distance was measured during a 1.0 μs molecular dynamics (MD) simulation. Utilizing a 1 μs MD simulation enhances the likelihood of capturing the full receptor conformational change stabilized by ligand binding, which is believed to require several hundred nanoseconds.^44^

**Figure 4:**
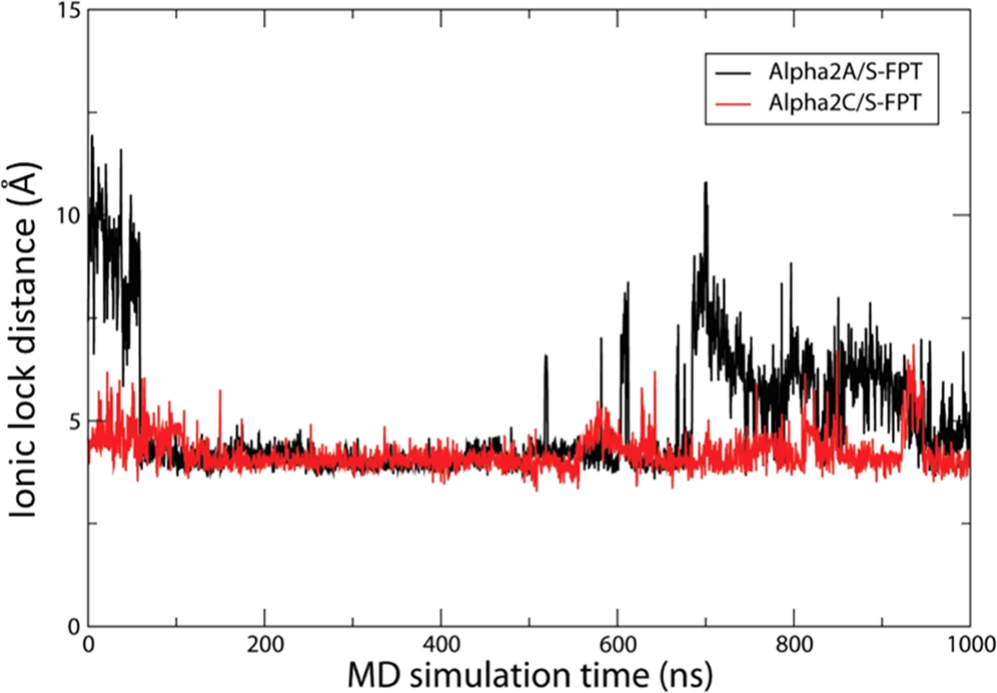
Molecular dynamics simulations showing the ionic lock distance, the distance between R3.50 and D6.30, when FPT is bound. This bond is engaged in α2CR, while it is broken in α2AR, supporting the observed *in vitro* profile.

For the α2AR bound to *S*-FPT (Figure 4, black distance line), the distance between R3.50 and D6.30 increases over time (e.g., 700–1000 ns), suggesting that the ionic bond breaks, as would occur for ligand agonist activity. For the α2CR bound to *S*-FPT (Figure 4, red distance line), the distance between R3.50 and D6.30 essentially remains constant over the 1.0 μs MD simulation, suggesting that the ionic bond remains intact, as would occur for ligand inverse agonist activity.

### Synthesis of 5-SATs

**Scheme 1:**
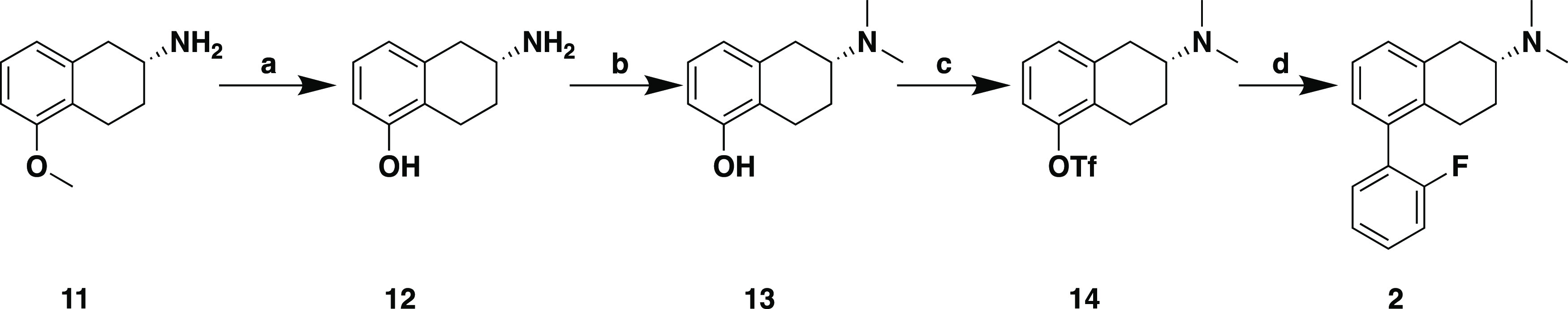
Chiral Synthesis of *R*-2′-F-5-PAT (FPT) Reagents and conditions. (a) HBr, 130 °C, 3.5 h; (b) CH_2_O, MeOH, reflux, 2 h then NaBH_4_, 0 °C to rt, 3 h; (c) *N*-(2-pyridyl)-bis(trifluoromethanesulfonimide), DIPEA, CH_2_Cl_2_, 0 °C to rt, 20 h; (d) 2-fluorophenylboronic acid, Pd(PPh_3_)_4_, K_2_CO_3_, 1,4-dioxane, 120 °C, 6 h.

The syntheses of analogues (*S*)-**2** and (*R*)- or (*S*)-**1–3** and **5–10** were reported previously.^45^ A stereoselective synthesis was utilized here to obtain (*R*)-**2** (Scheme 1). (*R*)-5-Methoxy-1,2,3,4-tetrahydronaphthalen-2-amine HCl **11** was O-demethylated using aqueous HBr to give adduct (*R*)-**12**. Reductive amination followed by sodium borohydride reduction gave intermediate (*R*)-**13**, which was reacted with *N*-(2-pyridyl)-bis(trifluoromethanesulfonimide) to form (*R*)-**14**. The triflate (*R*)-**14** was coupled with 2-fluorophenylboronic acid via Suzuki–Miyaura coupling to give (*R*)-**2** in a consistent 54% yield. Free base (*R*)-**2** was converted to corresponding HCl salt using 2 M HCl in ether.

**Scheme 2:**
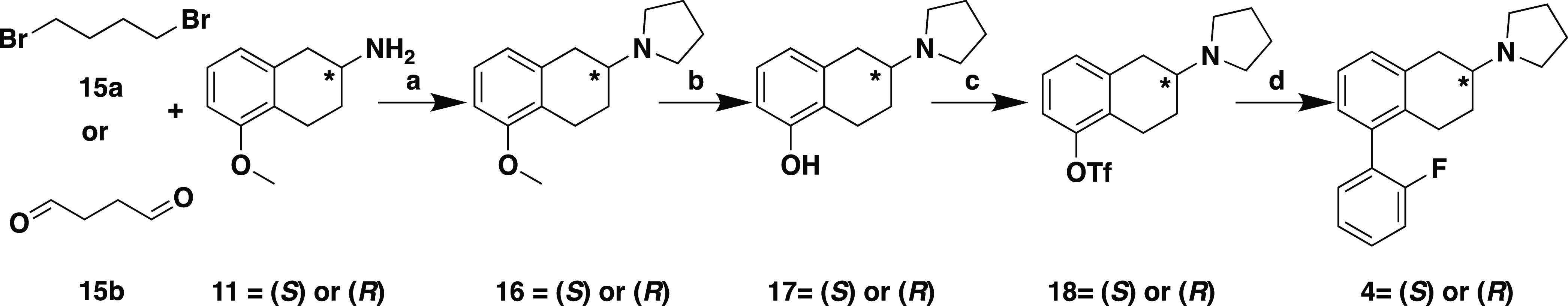
Chiral Synthesis of (2-Pyrrolidinyl)-2′-F-5-PAT (PFPT) Reagents and conditions. (a) 1,4-dibromobutane, TEA, ACN, reflux 4 h, then 40 °C, 30 min, or, 1,4-butandial, CH_3_COOH, NaBH_3_CN, ACN, rt, 4 h; (b) HBr,130 °C, 3.5 h; (c) *N*-(2-pyridyl)-bis(trifluoromethanesulfonimide), DIPEA, CH_2_Cl_2_, 0 °C to rt, 20 h; (d) 2-fluorophenylboronic acid, Pd(PPh_3_)_4_, K_2_CO_3_, 1,4-dioxane, 120 °C, 6 h.

A similar stereoselective route (Scheme 2) was developed to synthesize (*S*)-**4** and (*R*)-**4**. (*S*)-5-Methoxy-1,2,3,4-tetrahydronaphthalen-2-amine HCl **11** was reacted with 1,4-dibromobutane **15a** to form (*S*)-**16**, or, underwent a reductive amination of (*R*)-5-methoxy-1,2,3,4-tetrahydronaphthalen-2-amine HCl **11** with 1,4-butandial **15b** in the presence of sodium cyanoborohydride and acetic acid gave (*R*)-**16**. The 5-methoxy group was removed with aqueous HBr to form alcohol (*R*)- or (*S*)-**17**, and the corresponding triflates (*R*)- or (*S*)-**18** were formed in ∼16% yield using *N*-(2-pyridyl)-bis(trifluoromethanesulfonimide). (*R*)- or (*S*)-**18** were reacted with 2-fluorophenylboronic acid via Suzuki–Miyaura coupling to form (*R*)- or (*S*)-**4** in ∼57% yield. Free base (*R*)- or (*S*)-**4** were converted to the corresponding HCl salts using 2 M HCl in ether.

## Discussion

The results of these studies indicate that the 5-SAT chemotype has high affinity for the α2AR and the α2CR when appropriately substituted. The lower affinity of dipropylamine-substituted 5-SATs (DPAT, DCPT) compared to the dimethylamine (CPT, FPT) and pyrrolidinyl (PFPT) compounds, likely is due to the relatively restricted space around the D3.32 residue conserved in both receptors (Figure 3). Another important finding is that in contrast to approved α2R agonists such as lofexidine (as well as clonidine and guanfacine), which are agonists at both α2ARs and α2CRs, certain 5-SATs (**1–4**, **7**) have uniquely selective functional activity at α2Rs, i.e., they are agonists at the α2AR but inverse agonists at the α2CR. Molecular modeling results suggest the 5-SATs can stabilize functionally distinct α2AR and α2CR conformations, and the integration of our *in vitro* experimental data with computational molecular docking and dynamics data provides insight into the molecular basis for α2R conformational selectivity.

For example, at the α2AR, the 2′-fluorophenyl moiety of FPT (as well as the dichlorophenyl moiety of lofexidine) docks close to the hydroxyl moieties of S5.42 and Y6.55. This observation is consistent with docking results for lofexidine at α2AR models based on the crystal^31^ and cryo-EM^38^ structures, which showed that strong interactions with these residues are necessary for receptor activation. In our studies, lofexidine is a high-efficacy α2AR agonist, and with FPT (and the other 2′-halophenyl-substituted 5-SATs CPT and PFPT) possessing the highest efficacy of the 5-SATs, which suggests that the halogen bonding interaction for lofexidine and FPT with the α2AR S5.42 and Y6.55 residues is productive to realize physiologically relevant^32,33,46^ agonism.

At the α2CR, however, FPT (along with the CPT and PFPT) are inverse agonists, whereas lofexidine is an agonist like at the α2AR. The FPT dock at α2CR shows that the 2′-fluorophenyl moiety orients far from the S5.42 and Y6.55 residues. This appears to be due to the nonconserved ECL2 residue^30^ L45.52, which seems to sterically hinder this interaction, impeding the ligand′s ability to orient further into the binding pocket. In contrast, the lofexidine dock indicates that there is still close interaction between its dichlorophenyl moiety and the α2CR residues S5.42 and Y6.55, as is the case at the α2AR. In addition, at the α2CR model, the 2′-fluorophenyl moiety of FPT can interact with C3.36, apparently forming a halogen bond that may further compromise the ability of the 2-fluorophenyl moiety to interact with S5.42 and Y6.55. The interaction between C3.36 and the FPT 2′-fluorophenyl moiety also may impact the ability of R3.50 to disengage from presumed ionic bonding with D6.30, thus keeping the α2CR in an inactivated state. This proposal is strengthened by MD results that show the distance between R3.50 and D6.30 is not increased over the 1 μs experiment. Related, we considered that the observed increase in cAMP formation produced by FPT interaction with the α2CR may be due to FPT stabilization of a α2CR conformation that couples to Gα_s_ rather than Gα_i_, a phenomenon that has been reported in *in vitro* assays.^31,47^ However, the MD results indicating that the R3.50 and D6.30 distance did not change over 1 μs suggest that no conformational change has occurred, and thus an inverse agonist conformation of α2CR is stabilized by FPT, as opposed to the α2CR coupling to Gα_s_. Further, work done when the α2AR was crystallized^31^ suggests that interaction with two residues on ICL2 (I34.51 and K34.56) may predict Gs coupling. Ligand interactions did not impact these residues here.

The 5-PAT and NAP analogues also demonstrate α2R selective functional activity like the 2′-halophenyl 5-SATs (**2–4**), i.e., agonist activity at the α2AR but inverse agonism at the α2CR. We note however that 5-PAT and NAP, which cannot realize halogen binding with S5.42 and Y6.55, have lower α2AR agonist potency and efficacy compared to the 2′-halophenyl-substituted 5-SATs (**2–4**). Moreover, the 5-PAT and NAP analogues are significantly less potent as α2CR inverse agonists as compared to the 2′-halophenyl analogues, consistent with their inability to form halogen interactions with C3.36, perhaps compromising their ability to prevent the ionic lock from breaking.

Comparing the docks of FPT and lofexidine at the α2AR provides inference into the higher efficacy observed for lofexidine. Notably, the imidazoline nitrogen of lofexidine, which is presumably protonated at physiological pH, is observed to be closer in proximity to D3.32, ∼1 Å closer than the 2-amino nitrogen of FPT. This would likely induce a stronger ionic bond between the conserved D3.32 residue and the protonated nitrogen of lofexidine, sustaining the bond and possibly enhancing the stability of the active state conformation, perhaps correlating to more pronounced efficacy. Also, molecular docking results indicate that the oxygen linker of lofexidine appears to interact with α2AR F6.51 (not shown in Figure 3), which may stabilize the aromatic cage region of the receptor,^31^ similar to results reported for the nitrogen linker of clonidine.^48^

The partial agonist efficacy of FPT at the α2AR may be beneficial in a therapeutic context. For example, partial agonists may avoid agonist-induced receptor desensitization and/or downregulation, which could potentially minimize chronic and/or rebound effects that are observed with full-efficacy α2AR agonists,^49−52^ including lofexidine.^53^ Full-efficacy agonism can correlate with receptor desensitization, as has been observed in other Class A GPCRs such as the 5HT_2C_ receptor^54^ and the μ-opioid receptor.^55^ Studies are in progress to determine if 5-SATs cause α2R desensitization.

As noted, it has been shown that activation of the α2CR may be detrimental, particularly regarding cognitive measures.^23−25,56^ For example, the beneficial effects on cognition triggered by α2R agonists such as guanfacine have been shown to be mediated solely by α2AR,^27^ while antagonism of the α2CR has been shown to enhance episodic memory in human clinical trials.^28^ Thus, the conformationally selective profile demonstrated by 2′-halophenyl 5-SATs may demonstrate therapeutic potential in a variety of different contexts and serves as valuable probes in future *in vivo* studies.

In conclusion, here we report a new class of high affinity α2R ligands, which includes the previously reported compound FPT that was shown to improve repetitive behaviors, social behaviors, anxiety behaviors, and ameliorate seizures in mouse models of ASD.^32^ The neurotherapeutic effects of FPT should now be reexamined and reinterpreted in light of the new findings reported here that it is a α2AR agonist with α2CR inverse agonist activity. To the best of our knowledge, FPT and other 5-SATs reported here are the only known monovalent small molecules that activate the α2AR but inactivate the α2CR. Molecular modeling and MD results suggest that the unique α2R functional activity of 5-SATs results from conformationally selective interactions at the α2AR and α2CR. Data reported here will aid in our understanding of how ligands interact with the receptor and enhance future drug discovery, targeting α2R. Further synthetic efforts will be undertaken to expand on the SAR and supplement the conformationally selective profile.

## Methods

### Compounds

Compounds evaluated were of the 5-substituted-2-aminotetralin chemotype. Single enantiomers were generally obtained via the chiral synthetic scheme reported here; otherwise, enantiomers were resolved by chiral stationary phase high-performance liquid chromatography and converted to hydrochloride salts using previously published synthetic methods.^45^ Lofexidine hydrochloride and yohimbine were acquired from Sigma-Aldrich (St. Louis, MO). [^3^H]Rauwolscine was acquired from PerkinElmer (Waltham, MA).

### Cell Culture and Transfection

HEK293 (ATCC: CRL-1573) cells were cultivated in minimum essential media (MEM) (Corning) supplemented with 10% fetal bovine serum (FBS) to encourage cell growth and 1% penicillin/streptomycin. Cells were grown in 10-cm plates and allowed to reach log growth phase (equivalent to about 70–90% confluency) before transfection. A transfection cocktail containing 3 mL of Opti-MEM (Gibco, Waltham, MA), 10 μg of cDNA, and 30 μL of linear polyethyleneimine “max” (molecular weight ∼ 40 000 g/mol, Polysciences Inc., Warrington, PA) was added to each plate and then incubated with an additional 3 mL of supplemented MEM for 48 h. Human wild-type α2-adrenergic GPCR clones, encoded in a pcDNA3.1(+) vector, were obtained from the cDNA Resource Center (Bloomsburg, PA). Cells were transiently transfected to express the α2AR or α2CR, and membranes were isolated for use in radioligand binding assays. For functional assays, mouse connective-tissue cells stably expressing the human wild-type α2AR (ATCC: CRL-11180) or α2CR (ATCC: CRL-11181) were utilized.

### Radioligand Binding Experiments

Compound affinities were determined based on a modified version of a procedure that has been used in our laboratory and previously described.^45,57^ Cell membrane isolates were obtained following transfection procedure via a series of three 10-min, 12000*g* centrifugations homogenized via a tissue grinder. Saturation and competitive radioligand displacement assays conducted in 96-well assay plates were performed using membrane isolates. The bicinchoninic acid (BCA) protein assay (Thermo Scientific) kit was used to quantify protein expression. The dissociation constant (*K*_D_) of [^3^H]rauwolscine and receptor density labeled (*B*_max_) were determined from saturation binding experiments using concentrations of [^3^H]rauwolscine from 0.1–10 nM in triplicate; *K*_D_ = 1.07 ± 0.02 nM for the α2AR and 0.47 ± 0.02 nM for the α2CR; *B*_max_ = 13.1 ± 0.214 pmol/mg for the α2AR and 10.3 ± 0.714 pmol/mg for the α2CR.

Compounds were assessed across 10–12 concentrations in half-log units from 10 pM to 100 μM, with the median concentration being the approximate IC_50_ for displacement of [^3^H]rauwolscine at its *K*_D_ for the α2AR or α2CR. Each concentration was assessed in quadruplicate, and each competitive binding assay was performed in at least three distinct replicates. Nonspecific binding was determined using 10 μM clozapine. Data were analyzed using the nonlinear regression function “One-site-Fit *K*_i_” in Prism, wherein the *K*_i_ was calculated from the experimentally derived IC_50_ via the Cheng–Prusoff equation^58^
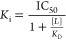


[*L*] is the concentration of [^3^H]rauwolscine and *K*_D_ at each receptor was determined above.

### Cyclic Adenosine Monophosphate Accumulation Assays

Receptor-mediated inhibition or stimulation of adenylyl cyclase was measured in cells stably expressing either α2AR or α2CR a LANCE Ultra cAMP assay kit (PerkinElmer, Waltham, MA), a time-resolved fluorescence energy transfer (TR-FRET) immunoassay, in a manner consistent with the manufacturer’s recommendations. Minor modifications were made to optimize conditions. Assays were conducted in 384-well plate format using ∼2000 cells/μL dilutions of compound prepared in 8–10 concentrations of ligand ranging from 10 pM to 100 μM, intended to have the median concentration to be the approximate EC/IC_50_ of the ligand (estimated based on *K*_i_ values). Each concentration of ligand was applied in triplicate to the cells. A forskolin concentration of 600 nM was applied to the cells to generate a baseline, with the concentration chosen to accommodate the assay window. Cells were incubated for 30 min at 37 °C (α2AR assays) and for 2 h at room temperature (α2CR assays); time was chosen based on optimization-determined ideal conditions and activation kinetics.^59^ Cells were then lysed and incubated with Eu-chelated tracer and Ulight anti-cAMP for 1 h. Fluorescence in the lysed cells was measured at 665 nm after stimulation at 615 nm. In addition, 11 concentrations of cAMP ranging from 10 pM to 1 μM in half-log units and a blank (assay buffer) were used to establish a standard curve. The concentration of cAMP present in each well was interpolated from this curve using the raw fluorescence value. Data presented is normalized as a percentage of the lofexidine-mediated inhibition of cAMP production, wherein 100% is defined as the maximum activation by lofexidine and 0% is forskolin-stimulated baseline (basal signaling).

### Molecular Modeling

#### Molecular Docking

Docks were developed via a modified version of a procedure that has been used in our laboratory and previously described.^57^ Three-dimensional ligands were assembled in Maestro (Schrodinger, New York, NY) and optimized via an *ab initio* quantum chemistry method at the HF/6-31G* level, followed by single-point energy calculations of molecular electrostatic potential for charge fitting with Gaussian 16.^60^ These docks were formulated using the solved crystal structures for α2AR (PDB: 6KUX) and α2CR (PDB: 6KUW), each reflecting the receptors’ inactive state, with omissions of sideloops and chains added and recapitulated using BIOVIA’s Discovery Studio 2017 (Dassault Systems, Waltham, MA). No sidechains were missing within the binding sites of the crystal structures used for the docks. The following residues composed the binding sites: PDB 6KUX (VAL86, SER90, TYR109, ASP113, VAL114, LYS117, THR118, ILE190, VAL197, SER200, CYS201, SER204, TRP387, PHE390, PHE391, TYR394, PHE412, TYR416) and PDB 6KUW (VAL104, SER108, TYR127, LEU128, ASP131, VAL132, CYS135, GLY203, LEU204, SER218, TRP395, PHE398, PHE399, TYR402, PHE419, PHE423, TYR427).

The ligands were docked into the binding sites in the receptors using induced-fit-docking (IFD) simulations^61^ (Schrödinger, Inc.). The default parameters were used for IFD simulations. The residues within 5 Å of ligand poses were selected for side chain optimization by prime refinement. The XP scores were used for ranking of the ligand poses, and top 20 poses of docked ligand were saved for visual inspection and selection. The poses of docked ligands with the lowest docking XP score were selected as predicted poses.

#### Molecular Dynamics

Protonation states of the titratable residues in α2AR and α2CR were calculated at pH = 7.4 via the use of the H++ server (http://biophysics.cs.vt.edu/). The ligand–receptor complexes identified in the molecular docks were inserted into a simulated lipid bilayer composed of POPC/POPE/cholesterol (2:2:1)^62^ and a water box using the CHARMM-GUI Membrane Builder webserver (http://www.charmm-gui.org). Sodium chloride (150 mM) as well as neutralizing counter ions were applied to the systems. The PMEMD.CUDA program of AMBER 16 was used to conduct MD simulations. The Amber ff14SB, lipid17, and TIP3P force fields were used for the receptors, lipids, and water, respectively. The parameters of lofexidine and (*S*)-FPT were generated using a general AMBER force field by the Antechamber module of AmberTools 17. The partial charge was determined via a restrained electrostatic potential charge-fitting scheme by *ab initio* quantum chemistry at the HF/6-31G* level.^60^ Coordinate files and system topology were established using the tleap module of Amber. The systems were energetically minimized by 500 steps (with a position restraint of 500 kcal·mol^–1^·Å^–2^) followed by 2000 steps (without position restraint) using the steepest descent algorithm. Heat was then applied to the systems to drive the temperature from 0 to 303 K using Langevin dynamics with a collision frequency of 1 ps^–1^. Receptor complexes were position-restrained using an initial constant force of 500 kcal·mol^–1^·Å^–2^ during the heating process, subsequently diminished to 10 kcal·mol^–1^·Å^–2^, allowing the lipid and water molecules free movement. Before the MD simulations, the systems underwent 5 ns equilibration. Then, a total of 100–1000 ns of MD simulations were conducted, with coordinates being saved every 100 ps for analysis. The simulations were conducted in an isothermal and isobaric nature, with the pressure maintained using an isotropic position scaling algorithm with the pressure relaxation time fixed at 2 ps. Long-range electrostatics were calculated by a particle mesh Ewald method with a 10 Å cut-off.^63^

### Chemistry

All commercially available reagents and solvents were purchased from Fisher Scientific or Sigma-Aldrich and used without purification. All reactions were performed under an inert atmosphere of anhydrous nitrogen. Flash column chromatography was performed using Agela Technologies 230–400 mesh silica gel. Analytical thin-layer chromatography (TLC) was carried out on Merck silica gel 60 F_254_ plates. Final compounds were used as their corresponding HCl salts utilizing 2 M HCl ester from Fisher Scientific, as noted below. All NMR spectra were recorded by a Varian 500 MHz or Bruker Avance 500 MHz NMR in CDCl_3_ and are expressed as chemical shift (δ) values in parts per million (ppm). Coupling constants (*J*) are presented in Hertz. Abbreviations used in the reporting of NMR spectra include s = single, bs = broad singlet, d = doublet, t = triplet, sext = sextet, oct = octet, and m = multiplet. High-resolution mass spectrometry (HRMS) was performed with Waters Q-TOF Ultima ESI instrument using time of flight (TOF-MS) and electron spray ionization (ESI).^45^

#### (*R*)-6-Amino-5,6,7,8-tetrahydronaphthalen-1-ol (**[2R]-12**)

(*R*)-5-Methoxy-1,2,3,4-tetrahydronaphthalen-2-amine HCl **(2*****R*****)-11** (100 mg, 0.480 mmol, 1.00 equiv) was suspended in 1 mL of 48% aq HBr (9.6 mmol, 20 equiv). The reaction was stirred under reflux for 3.5 h. It was cooled to room temperature, the solvent was evaporated *in vacuo* with NaOH in the receiving flask, and the water bath was set to 60 °C. MeOH was continually added to aid in the evaporation process. Once completely dry, the residue was saturated under vacuum overnight. No further purification was done yielding 120 mg of a light brown solid. TLC eluents used were 9:1 DCM/MeOH.

#### (*R*)-6-(Dimethylamino)-5,6,7,8-tetrahydronaphthalen-1-ol (**[2R]-13**)

(*R*)-6-Amino-5,6,7,8-tetrahydronaphthalen-1-ol **(2*****R*****)-12** (120 mg, 0.730 mmol, 1.00 equiv) was dissolved in 5 mL of MeOH, and formaldehyde (0.2 mL, 7.30 mmol, 10.0 equiv) was added. The reaction was stirred at 130 °C under reflux for 2 h. The reaction was cooled on ice, and sodium borohydride (170 mg, 4.50 mmol, 6.00 equiv) was added slowly. The reaction was cooled to room temperature and stirred for 3 h. Saturated sodium bicarbonate (15 mL) was added and extracted with EtOAc (2 × 15 mL). The organic layers were combined and dried over sodium sulfate. No further purification was done, yielding 50.0 mg of a clear oil. TLC eluents used were 95:5 dichloromethane/MeOH.

#### (*R*)-6-(Dimethylamino)-5,6,7,8-tetrahydronaphthalen-1-yl-trifluoromethanesulfonate (**[2R]-14**)

(*R*)-6-(Dimethylamino)-5,6,7,8-tetrahydronaphthalen-1-ol **(2*****R*****)-13** (50.0 mg, 0.30 mmol, 1.00 equiv) was dissolved in 4.5 mL of anhydrous DCM. *N*-(2-Pyridyl)-bis(trifluoromethanesulfonimide) (240 mg, 0.70 mmol, 2.33 equiv) was added to the reaction. The reaction was cooled to −78 °C using a dry ice/acetone bath and stirred for 5 min to allow the solution to cool. *N*,*N*-Diisopropylethylamine (235 μL, 1.80 mmol, 6.00 equiv) was added to the solution dropwise using a syringe. The solution was slowly warmed to room temperature and stirred for 20 h. After 20 h, 25 mL of saturated aqueous ammonium chloride was used to quench the reaction on ice. The aqueous layer was extracted with dichloromethane (3 × 20 mL). The organic layers were combined and dried using sodium sulfate and concentrated *in vacuo*. No further purification was done, yielding 100 mg of the product as a light red oil. TLC eluents used were 5:5:1 hexanes/EtOAc/TEA.

#### (*R*)-5-(2-Fluorophenyl)-*N*,*N*-dimethyl-1,2,3,4-tetrahydronaphthalen-2-amine (**[2R]-2**)

(*R*)-6-(Dimethylamino)-5,6,7,8-tetrahydronaphthalen-1-yl-trifluoromethanesulfonate **(2*****R*****)-14** (0.1 g 0.3 mmol) was dissolved in 1.5 mL of anhydrous 1,4-dioxane. 2-Fluorophenylboronic acid (65 mg, 0.465 mmol,1.50 equiv) was added to the reaction. The solution was degassed for 30 min. Pd(PPh_3_)_4_ (18 mg, 0.0155 mmol, 0.05 equiv) was added to the solution. KPO_3_ (118 mg, 0.558 mmol, 1.80 equiv) and KBr (41 mg, 0.341 mmol, 1.10 equiv) were added to the solution. The flask was fitted with a reflux condenser and heated at 120 °C for 6 h. The reaction was cooled, and the solvent was evaporated *in vacuo*. The residue was resuspended using 30 mL of EtOAc and 30 mL of water. The aqueous layer was extracted with EtOAc (2 × 15 mL). The organic layers were combined and washed with saturated aqueous sodium chloride (2 × 20 mL), dried with sodium sulfate, and concentrated *in vacuo*. Purification was done by flash chromatography 2:1:0.1 hexanes/EtOAc/TEA to yield 80 mg of the product as a clear oil. TLC eluents used were 5:5:1 hexanes/ethyl acetate/triethylamine. The oil was converted to the corresponding HCl salt (43.2 mg, 54%). ^1^H NMR (500 MHz; CDCl_3_): δ 1.88 (s, 1H), 2.43 (t, *J* = 3.1 Hz, 1H), 2.71 (s, 1H), 2.85 (s, 7H), 3.29–3.18 (m, 1H), 3.46–3.37 (m, 1H), 3.60–3.50 (m, 1H), 7.19 (d, *J* = 7.3 Hz, 2H), 7.21 (d, *J* = 7.5 Hz, 2H), 7.29 (t, *J* = 5.4 Hz, 2H), 7.38 (m, 1H), 12.83 (s, 1H). ^13^C NMR (500 MHz; CDCl_3_): δ 131.85, 131.24, 129.59, 129.53, 129.23, 128.80, 126.55, 124.29, 115.65, 62.40, 39.40, 29.93, 26.68, 26.23, 23.90. ^19^F NMR (500 MHz; CDCl_3_): δ −114.58, −115.16. HRMS calcd C_18_H_20_FN for [M + H]^+^: 270.1658; found: 270.1723

#### 1,4-Butandial (**15b**)

0.025 M HCl (3.4 mL, 0.08 mmol, 1.00 equiv) was added to 2,5-dimethoxytetrahydrofuran (1 g, 7.50 mmol, 93.75 equiv) and saturated at 0 °C for 16 h. The pH was adjusted to 6 using saturated aqueous sodium bicarbonate and diluted with water to afford a 1 M solution.

#### (*R*)-1-(5-Methoxy-1,2,3,4-tetrahydronaphthalen-2-yl)pyrrolidine (**[2R]-16**)

(*R*)-5-Methoxy-1,2,3,4-tetrahydronaphthalen-2-amine hydrochloride **(2*****R*****)-11** was converted to the corresponding free base. (*R*)-5-Methoxy-1,2,3,4-tetrahydronaphthalen-2-amine (250 mg, 1.40 mmol, 1.00 equiv) was dissolved in 10 mL of ACN. The prepared 1,4-butandial (7 mL, 7.00 mmol, 5.00 equiv) solution was added and stirred for 15 min at room temperature. NaBH_3_CN (190 mg, 3.00 mmol, 3.00 equiv) was added and stirred for 15 min at room temperature. Acetic acid (0.4 mL, 7.00 mmol, 5.00 equiv) was added and stirred for 4 h at room temperature. The reaction was quenched using 2 mL of 2 N NaOH or until pH 12. 10 mL of water was added and extracted with EtOAc (2 × 30 mL). Organic fractions were combined and washed with 2 N NaOH (2 × 15 mL), dried over sodium sulfate, and concentrated *in vacuo*. No further purification was done yielding 220 mg of a white oil. TLC eluents used were 20% TEA in EtOAc.

#### (*S*)-1-(5-Methoxy-1,2,3,4-tetrahydronaphthalen-2-yl)pyrrolidine (**[2S]-16**)

(*S*)-5-Methoxy-1,2,3,4-tetrahydronaphthalen-2-amine hydrochloride (80 mg, 0.46 mmol, 1.00 equiv) was dissolved in 3.5 mL of ACN. 1,4-Dibromobutane **15a** (61 μL, 0.51 mmol, 1.11 equiv) and TEA (212 μL, 15.5 mmol, 33.70 equiv) were added to the reaction and stirred at reflux for 4 h. The reaction was quenched on ice using water. The aqueous layer was extracted with EtOAc (2 × 15 mL). Organic fractions were combined and dried over sodium sulfate, and concentrated *in vacuo*, yielding 87 mg. No further purification was done.

#### General Methoxy Removal Procedure for analogues (**2*****R***)**-17**, (**2*****S***)**-17** (Scheme 2)

The corresponding intermediate **(2*S*)-16, (2*R*)-16** (1 equiv) was suspended in 48% aq HBr (40 equiv). The reaction was stirred at 130 °C under reflux for 3.5 h. The reaction was brought to room temperature and ∼5 mL of MeOH was added and evaporated *in vacuo* with NaOH pellets in the receiving flask and the water bath set to 60 °C. MeOH was continuously added to aid in evaporation. No further purification was done.

#### (*R*)-6-(Pyrrolidin-1-yl)-5,6,7,8-tetrahydronaphthalen-1-ol (**[2R]-17**)

Obtained from **(2*****R*****)-16** (0.95 mmol) yielding 0.206 g as a brown solid.

#### (*S*)-6-(Pyrrolidin-1-yl)-5,6,7,8-tetrahydronaphthalen-1-ol (**[2S]-17**)

Obtained from **(2*****S*****)-16** (2.5 mmol) yielding 0.412 g as a brown solid.

#### General Triflation Procedure for Analogues (**2*****R***)**-18**, (**2*****S***)**-18** (Scheme 2)

The corresponding intermediate **(2*R*)-17, (2*S*)-17** was dried *in vacuo* overnight. The corresponding intermediate **(2*R*)-17, (2*S*)-17** (1 equiv) was dissolved in anhydrous DCM. *N*-(2-Pyridyl)-bis(trifluoromethanesulfonimide) (1.5 equiv) was added at room temperature. The reaction was cooled to −78 °C using a dry ice/acetone bath and stirred for 5 min. *N,N*-Diisopropylethylamine (3 equiv) was added dropwise. The reaction was warmed to room temperature and stirred for 20 h. The reaction was quenched on ice using saturated aqueous ammonium chloride. The aqueous layer was extracted with DCM (3×). Organic fractions were combined and dried over sodium sulfate and concentrated *in vacuo.* Purification was done by flash chromatography (5:5:1 hexanes/EtOAc/TEA). TLC eluents used were 5:5:1 hexanes/EtOAc/TEA.

#### (*R*)-6-(Pyrrolidin-1-yl)-5,6,7,8-tetrahydronaphthalen-1-yl-trifluoromethanesulfonate (**[2R]-18**)

Obtained from **(2*****R*****)-17** (0.95 mmol) yielding 50 mg (15%) as a light-yellow oil. ^1^H NMR (500 MHz; CDCl_3_): δ 1.44 (m,1H), 1.60 (q, *J* = 10.0 Hz, 3H), 1.97 (s,1H), 2.16 (d, *J* = 12.1 Hz, 1H), 2.39 (t, *J* = 9.0 Hz, 1H), 2.63 (t, *J* = 13.5 Hz, 4H), 2.76 (t, *J* = 10.3 Hz, 1H), 2.98 (q, *J* = 16.2 Hz, 2H), 6.99 (d, *J* = 7.8 Hz, 1H), 7.04 (d, *J* = 7.4 Hz, 1H), 7.10 (t, *J* = 7.6 Hz, 1H) ^19^F NMR (500 MHz; CDCl_3_): δ −73.903, −74.014, −74.073

#### (*S*)-6-(Pyrrolidin-1-yl)-5,6,7,8-tetrahydronaphthalen-1-yl-trifluoromethanesulfonate (**[2S]-18**)

Obtained from **(2*****S*****)-17** (0.45 mmol) yielding 520 mg (16%) as a light-yellow oil. ^1^H NMR (500 MHz; CDCl_3_): δ 1.70 (m, 1H), 1.87 (m, 3H), 2.27 (m, 1H), 2.46 (m, 1H), 2.58 (m, 1H), 3.158–2.67 (m, 8H), 6.59 (d, *J* = 7.9 Hz, 1H), 6.68 (d, *J* = 7.5 Hz, 1H), 6.99 (t, *J* = 7.67 Hz, 1H).^19^F NMR (500 MHz; CDCl_3_): δ −73.534, −73.993.

#### General Suzuki Coupling Conditions for Analogues **(2*R*)-4**, **(2*S*)-4**

The corresponding intermediate **(2*****R*****)-18, (2*****S*****)-18** was dried overnight *in vacuo*. The corresponding intermediate **(2*****R*****)-18, (2*****S*****)-18** (1 equiv) was dissolved in anhydrous 1,4-dioxane. 2-Fluorophenylboronic acid (4 equiv) was added to the reaction. The solution was degassed with N_2_ for 30 min and Pd(PPh_3_)_4_ (0.1 equiv) was added along with KPO_3_ (1.5 equiv) and KBr (1.13 equiv). The reaction was heated to 120 °C under reflux for 6 h. The reaction was cooled, and the solvent was evaporated *in vacuo*. The reaction was resuspended using EtOAc and water. The aqueous layer was extracted with EtOAc (2×). The organic fractions were combined and washed with saturated aqueous sodium chloride and dried over sodium sulfate and concentrated *in vacuo*. Purification was done by flash chromatography (5:1:0.1 hexanes/EtOAc/TEA to 4:2:0.1 hexanes/EtOAc/TEA). TLC solvents used were 5:5:0.1 hexanes/EtOAc/TEA.

#### (*R*)-1-(5-(2-Fluorophenyl)-1,2,3,4-tetrahydronaphthalen-2-yl)pyrrolidine (**(2*R*)-4**)

Obtained from **(2*R*)-18** (0.14 mmol) yielding 38 mg as a light-yellow oil. The oil was converted to the corresponding HCl salt to yield a white solid (23 mg, 56%). ^1^H NMR (500 MHz; CDCl_3_): δ 7.29 (oct, *J* = 3.8 Hz, 1H), 7.16 (m, 4H), 7.04 (d, *J* = 7.0 Hz, 2H), 3.98–3.81 (m, 1H), 3.64 (t, *J* = 4.8 Hz, 2H), 3.46 (bs, 3H), 3.32 (m, 1H), 3.09 (d, *J* = 5.7 Hz, 4H), 2.69 (m, 2H), 2.02 (s, 2H). ^13^C NMR (500 MHz; CDCl_3_): δ 134.22, 133,53, 131.48, 129.50, 129.18, 126.56, 124.31, 115.63, 67.79, 58.08, 31.95, 30.57, 29.71, 24.62, 23.98, 22.99. ^19^F NMR (500 MHz; CDCl_3_): δ −114.33, −115.25. HRMS calcd C_20_H_22_FN for [M + H]^+^: 296.1815; found: 296.1899.

#### (*S*)-1-(5-(2-Fluorophenyl)-1,2,3,4-tetrahydronaphthalen-2-yl)pyrrolidine (**(2*S*)-4**)

Obtained from **(2*****S*****)-18** (0.15 mmol) yielding 47 mg as a light-yellow oil. The oil was converted to the corresponding HCl salt to yield a white solid (25 mg, 58%). ^1^H NMR (500 MHz; CDCl_3_): 12.67 (s, 1H), 7.38 (sext, *J* = 4.4 Hz, 1H), 7.25 (t, *J* = 7.6 Hz, 2H), 7.17 (d, *J* = 7.3, 2H), 7.11 (d, *J* = 7.3 Hz, 2H), 3.97–3.79 (bs, 2H), 3.54 (m, 1H), 3.34 (t, *J* = 14.9 Hz, 2H), 2.93 (t, *J* = 8.1 Hz, 2H), 2.83–2.64 (m, 2 H), 2.32 (m, 3H), 2.09 (t, *J* = 11.2 Hz, 3 H). ^13^C NMR (500 MHz; CDCl_3_): δ 206.80, 131.39, 129.49, 129.42, 129.14, 128.60, 126.39, 124.38, 115.47, 62.02, 51.45, 32.64, 31.04, 26.04, 25.70, 23.42. ^19^F NMR (500 MHz; CDCl_3_): δ −114.62, −114.68. HRMS calcd C_20_H_22_FN for [M + H]^+^: 296.1815; found: 296.1833.

### Data Analysis and Exclusion

All data analysis was conducted using GraphPad Prism 9.0 (or higher), (San Diego, CA). Three or more replicates were performed per experiment, with three or more independent experiments being performed for each data point (listed in Tables 1 and 2). All results are reported as the mean ± SD for the indicated number of independent experiments. Binding data were fit using the “one-site” model, as two-curve fitting did not enhance the quality of fit and there were no indications in the data, such as biphasic curves or Hill slopes ≠ 1, to suspect multiple binding sites. Unpaired *t* tests and/or one-way analysis of variance (ANOVAs) with Tukey’s multiple-comparison test were used to evaluate the significance for *K*_i_ and EC/IC_50_ values, noted depending on the context.

For radioligand competitive displacement binding assay results, values exceeding total binding by 25% or greater were excluded, as well as in the cases where radioligand binding was incomplete (>30% remaining). These exclusions could be attributed to the experimenter error in optimization of assay conditions as well as situations where the quantity of radioligand used was too high relative to the *K*_D_. Additionally, a *K*_i_ value may be excluded in a data set of four or more replicates wherein the value of one replicate deviates by more than two standard deviations of the mean.

For functional assays, data points were excluded if values fell outside of the dynamic range of the assay (typically found to be from 0.5 to 5 nM of cAMP) or if they failed either of the following statistical outlier tests: *p* < 0.05 using the two-sided Grubbs test or *Q* = 1 using the ROUT test. The choice of outlier test was determined depending on the context such as the sample size and whether there were multiple suspected outliers present.

## Abbreviations

α2R: α2-adrenoreceptor
AC: adenylyl cyclase
ACN: acetonitrile
*B*_max_: maximum binding
CDCl_3_: deuterated chloroform
DCM: dichloromethane
DIPEA: *N*,*N*-diisopropylethylamine
*E*_max_: maximum effect
EtOAc: ethyl acetate
*I*_max_: maximum inhibition
*K*_D_: dissociation constant
*K*_i_: inhibition constant
MeOH: methanol
TEA: triethylamine
5-SAT: 5-substituted-2-aminotetralin
